# Effect of Storage Time on the Fermentation Quality, Bacterial Community Structure, and Metabolic Profiles of Jinmu Grain Grass Silage

**DOI:** 10.3390/microorganisms13091973

**Published:** 2025-08-23

**Authors:** Yaqin Tang, Qianqian Wang, Qiuyan Li, Yasong Wang, Lei Gong, Wenju Zhang, Junli Niu

**Affiliations:** 1 Animal Nutrition and Feed Science, College of Animal Science and Technology, Shihezi University, Shihezi 832000, China; t892585470@sina.com (Y.T.); 15864653585@163.com (Q.W.); qiuyanli2025@outlook.com (Q.L.); zhangwj1022@sina.com (W.Z.); 2 The Animal Husbandry and Aquatic Products Development Service Center of the First Division of Xinjiang Production and Construction Corps, Aral 832104, China; 18299088281@163.com (Y.W.); gonglei125@126.com (L.G.)

**Keywords:** Jinmu grain grass silage, storage time, nutritional composition, fermentation quality, bacterial community, metabolomics

## Abstract

This study aimed to investigate the effect of storage time on the fermentation quality, bacterial community structure, and metabolic profiles of Jinmu grain grass silage. It was ensiled in vacuum bags for 60 days. Samples were collected after 0, 3, 7, 15, 30, and 60 days of ensiling. Nutritional analysis revealed no significant differences in dry matter (DM), ether extract (EE), crude protein (CP), neutral detergent fiber (NDF), or acid detergent fiber (ADF) across storage periods (*p* > 0.05), but relative feeding value (RFV) significantly increased at 30 and 60 days (*p* < 0.05). Fermentation quality improved with prolonged storage, pH values declined to 4.01 at 60 days, while lactic acid (LA) and acetic acid (AA) increased significantly (*p* < 0.05). Butyric acid(BA) was undetected. 16S rDNA sequencing showed bacterial diversity (Chao1, Simpson, and Shannon indices) increased significantly at 30 and 60 days (*p* < 0.01); The relative abundance of *Lacticaseibacillus*, and *Amylolactobacillus* at 30 days were significantly higher than 0 and 60 days (*p* < 0.05); The relative abundance of *Stenotrophomonas*, *Serratia*, *Comamonas*, *GKS98_freshwater_group*, and *Sphingobium* at 60 days were significantly higher than 0 and 30 days (*p* < 0.05). Comprehensive targeted metabolomics identified 2958 metabolites. There were 256 differential metabolites shared by the comparison groups at 0, 30, and 60 days. The pathways for enrichment of differential metabolites mainly include plant hormone signal transduction, Histidine metabolism, arginine biosynthesis, etc. In conclusion, the storage time of Jinmu grain grass silage can enhance its fermentation quality by influencing microbial communities and metabolic pathways.

## 1. Introduction

As a global powerhouse in animal husbandry, China’s livestock sector is of significant international importance. However, the nation faces the persistent challenge of supporting a vast population with limited arable land, resulting in low per-capita grain availability [1]. This constraint has led to a critical shortage of feed resources, which now acts as a major bottleneck constraining the sustainable development of both the animal husbandry and feed industries in China [2]. Consequently, enhancing the development and utilization of alternative feed resources is imperative to ensure the nation’s food security and agricultural sustainability.

Jinmu grain grass is a plant species belonging to the genus *Pennisetum* of the Poaceae family. It is rich in nutrients, containing more than 18 essential amino acids and various vitamins that are beneficial to animal growth [3]. Moreover, its leaves have no burrs (steel hairs), which is conducive to digestion by livestock and poultry. Under the suitable climatic conditions for growing farmland in the western region, the annual output of fresh grass is 40 to 60 tons per mu. The Jinmu grain grass grown in Xinjiang can be harvested three times a year, usually from August to November [4]. The feed after ensiling is soft, juicy, fragrant, and palatable, and the nutritional value of the feed is improved, which is conducive to increasing the feed intake and digestion and utilization rate of livestock and poultry [5]. Therefore, ensiling processing can ensure the year-round supply of Jinmu grain grass for livestock and poultry and reduce nutrient loss during feed storage. In addition, the plants of Jinmu grain grass are tall, upright, and clustered, with well-developed root systems, wide adaptability, high stress resistance, fast growth rate, strong regenerative capacity, high yield, and are resistant to acid, high temperature, drought, and cold, as well as diseases and pests. They will not die within −30 °C and can be planted in most areas of China. Meanwhile, Jinmu grain grass is a typical four-carbon plant with a high photosynthetic rate, which can prevent soil erosion and is suitable for desertification control [6]. To sum up, Jinmu grain grass is an ideal feed resource for livestock and poultry.

However, the application of Jinmu grain grass as a feed resource has not been widely promoted, and there are few reports on the mechanism of its silage in enhancing nutritional value and the fermentation mechanism of the micro-ecosystem. Therefore, in this study, Jinmu grain grass was processed into silage to explore the changes in its nutritional components and silage quality at different storage times. The effects at 30 and 60 days of ensiling on the bacterial community of Jinmu grain grass were analyzed with 16Sr DNA sequencing, and comprehensive targeted metabolomics was conducted to screen the differential metabolites and metabolic pathways of each comparison group between 0, 30, and 60 days. By studying the nutritional composition, fermentation quality, microbial quantity, bacterial community structure, and metabolic characteristics of Jinmu grain grass at different storage times, the changes in its microbial community and metabolites were revealed. The aim of this study was to investigate the effects of storage time on the fermentation quality, bacterial community structure, and metabolic profiles of Jinmu grain grass silage. The results are expected to provide theoretical references for the promotion and application of Jinmu grain grass in livestock and poultry production and the healthy and sustainable development of China’s animal husbandry.

## 2. Materials and Methods

### 2.1. The Production of Jinmu Grain Grass Silage

The fresh Jinmu grain grass silage harvested in the Eighth Regiment of the First Division of Xinjiang Production and Construction Corps was manually cut into 1–2 cm sections, mixed evenly, and then placed in vacuum bags (30 cm × 50 cm) respectively. After that, they were taken back to the laboratory and placed in a shady and light-proof area.

### 2.2. Sample Collection of Jinmu Grain Grass Silage

Samples were taken by opening the vacuum bags after 0, 3, 7, 15, 30, and 60 days of ensiling, respectively. Five bags of samples were taken at each storage time. A total of 20 g of silage sample was weighed from each bag and placed in a 250 mL conical flask. A total of 180 mL of distilled water was added, and it was soaked in a 4 °C refrigerator for 24 h. First, the sample was filtered through 4 layers of coarse gauze, and then through a fine filter with filter paper to obtain the extract [7]. The remaining silage samples were weighed separately, dried, and sealed for future use.

### 2.3. Detection of Indicators

#### 2.3.1. Determination of Nutritional Composition

The nutritional quality indicators of silage mainly determine the contents of dry matter (DM), ether extract (EE), crude protein (CP), neutral detergent fiber (NDF), and acidic detergent fiber (ADF). Among them, DM, CP, and EE were determined respectively in accordance with the methods of the national standards GB/T 6435-2014 [8], GB/T 6432-2018 [9] and GB/T 6433-2006 [10], and the contents of NDF and ADF were determined with reference to the method of Van Soest et al. [7]. Calculate the relative feeding value (RFV) according to the following formula.

RFV = DMI × DDM/1.29(1)

DMI = 120/NDF(2)

DDM = 88.9 − 0.779 × ADF(3)

In the formula: DMI represents the voluntary dry matter intake (% BW), DDM represents the digestible dry matter content (% DM), and 1.29 is the parameter value of forage DMI × DDM.

#### 2.3.2. Determination of Silage Fermentation Quality

The pH value and NH_3_-N contents in silage extract were determined by using an acid meter and phenol-sodium hypochlorite colorimetric method, respectively. The lactic acid (LA), acetic acid (AA), propionic acid (PA), and Butyric acid (BA) contents were measured by high-performance liquid chromatography (Agi-Lent1260 HPLC system, Agilent Technologies, Santa Clara, CA, USA).

### 2.4. Bacterial Community Analysis

Total microbial DNA was extracted and PCR amplified from Jinmu grain grass samples after 0, 30, and 60 days of ensiling using the bacterial DNA kit (Omega Biotek, Norcross, GA, USA) [11]. The primers 338-F and 806-R were utilized to amplify the V3–V4 region of 16S rRNA on the Illumina MiSeq PE300 platform (Suzhou PANOMIX Biomedical Tech Co., Ltd., Suzhou, China). High-quality sequences were clustered into operational taxonomic units (OTUs) at 97% sequence similarity using UPARSE (version 7.1). The alpha-diversities (Chao1, Simpson, Shannon, and Goods coverage indexes) were performed using Mothur (version 1.30.1). The bacterial community structure was determined at the phylum and genus levels based on the Silva database (Release 132). Differential biomarkers between groups were identified via Linear Discriminant Analysis Effect Size (LEfSe) analysis (https://magic.novogene.com/customer/main#/homeNew, accessed on 8 July 2024). The sequence data had been submitted to the NCBI database under accession number PRJNA1293431.

### 2.5. Metabolomics Analysis

#### 2.5.1. Metabolite Extraction

An appropriate amount of samples of Jinmu grain grass was accurately weighed after 0, 30, and 60 days of ensiling into a 2 mL centrifuge tube, 600 µL MeOH (containing 2-Amino-3-(2-chloro-phenyl)-propionic acid (4 ppm)) was added, and it was vortexed for 30 s. Steel balls were added and placed in a tissue grinder for 60 s at 55 Hz. Room temperature ultrasound was conducted for 15 min. It was centrifuged for 10 min at 12,000 rpm and 4 °C. The supernatant was filtered using a 0.22 μm membrane and transferred into the detection bottle for LC-MS detection [12].

#### 2.5.2. Liquid Chromatography Conditions

The LC analysis was performed on a Vanquish UHPLC System (Thermo Fisher Scientific, Waltham, MA, USA). Chromatography was carried out with an ACQUITY UPLC ^®^ HSS T3 (2.1 mm × 100 mm, 1.8 µm) (Waters, Milford, MA, USA). The column was maintained at 40 °C. The flow rate and injection volume were set at 0.3 mL/min and 2 μL, respectively. For LC-ESI (+)-MS analysis, the mobile phases consisted of (B2) 0.1% formic acid in acetonitrile (*v*/*v*) and (A2) 0.1% formic acid in water (*v*/*v*). For LC-ESI (-)-MS analysis, the analytes were carried out with (B3) acetonitrile and (A3) ammonium formate (5 mM). Separation was conducted under the following gradient: 0~1 min, 10% B; 1~5 min, 10%~98% B; 5~6.5 min, 98% B; 6.5~6.6 min, 98%~10% B; 6.6~8 min, 10% B [13].

#### 2.5.3. Mass Spectrum Conditions

Mass spectrometric detection of metabolites was performed on Orbitrap Exploris 120 (Thermo Fisher Scientific, Waltham, MA, USA) with an ESI ion source. Simultaneous MS1 and MS/MS (Full MS-ddMS2 mode, data-dependent MS/MS) acquisition was used. The parameters were as follows: sheath gas pressure, 40 arb; aux gas flow, 10 arb; spray voltage, 3.50 kV and −2.50 kV for ESI(+) and ESI(−), respectively; capillary temperature, 325 °C; MS1 range, *m*/*z* 100–1000; MS1 resolving power, 60,000 FWHM [14].

#### 2.5.4. Metabolite Data Analysis

The raw data were converted to mzXML format using the MS convert tool (v3.0.8789). The R XCMS (v3.12.0) software was used for peak detection, filtering, alignment, and normalization. The selected metabolite data were then imported into Ropls software (v1.22.0) for principal component analysis (PCA), partial least squares discriminant analysis (PLS-DA), and orthogonal partial least squares discriminant analysis (OPLS-DA) [15]. Multivariate analysis identified metabolites with *p* < 0.05, VIP > 1, and FC > 2, and their data were identified using spectral databases such as HMDB, LipidMaps, and KEGG, providing detailed information on the differential metabolites. The differential metabolites were further analyzed for KEGG pathway enrichment, and pathways with impact > 0.1 and *p* < 0.05 were identified as differential metabolic pathways.

### 2.6. Data Analysis

Statistical analysis was conducted using SPSS 27.0.1, and charts were created using GraphPad Prism 10.1.2. The experimental data are presented as mean ± standard error ($\bar{x}\;\pm \;S$). One-way ANOVA was used, and Duncan multiple comparisons were applied post-hoc to the groups. *p* values < 0.05 indicate significant differences, and *p* values < 0.01 indicate extremely significant differences.

## 3. Results

### 3.1. The Influence of Different Storage Times on the Nutritional Composition of Jinmu Grain Grass

The dynamic changes of nutritional composition during the ensiling process of Jinmu grain grass are shown in Table 1. There were no significant differences in the contents of DM, EE, CP, NDF, and ADF among different storage times (*p* > 0.05). The RFV at 30 and 60 days was significantly higher than that after 0, 3, 7, and 15 days of ensiling (*p* < 0.05). There was no significant difference in RFV among 30 and 60 days, and 0, 3, 7, and 15 days of ensiling (*p* > 0.05).

### 3.2. The Influence of Different Storage Times on the Fermentation Quality of Jinmu Grain Grass

The dynamic changes in fermentation quality during the ensiling process of Jinmu grain grass are shown in Table 2. With the extension of storage time, the pH value showed a decreasing trend. It decreased to 4.01 in the later stage of ensiling (60 days), which was significantly lower than that in other stages except for 30 days of ensiling (*p* < 0.05). The content of NH_3_-N showed an increasing trend, and it was significantly higher after 60 days of ensiling than at other stages (*p* < 0.05). During the entire fermentation stage, the contents of LA and AA showed an increasing trend, and significantly increased after 30 and 60 days of ensiling (*p* < 0.05). No PA was detected at 0 days, but it was detected from 3 days to 60 days. Moreover, the PA content showed an increasing trend throughout the fermentation stage. The PA content at 60 days was significantly higher than that after 3, 7, and 15 days of ensiling (*p* < 0.05), but there was no significant difference compared with that at 30 days (*p* > 0.05). No BA was detected throughout the entire fermentation stage. It can be seen from the table that the accumulation rate of LA was higher than that of AA, which was conducive to the reduction of the pH value and the preservation of nutrients in silage.

### 3.3. The Influence of Different Storage Times on the Microbial Community of Jinmu Grain Grass

#### 3.3.1. Analysis of Microbial Community Diversity

**Figure 1:**
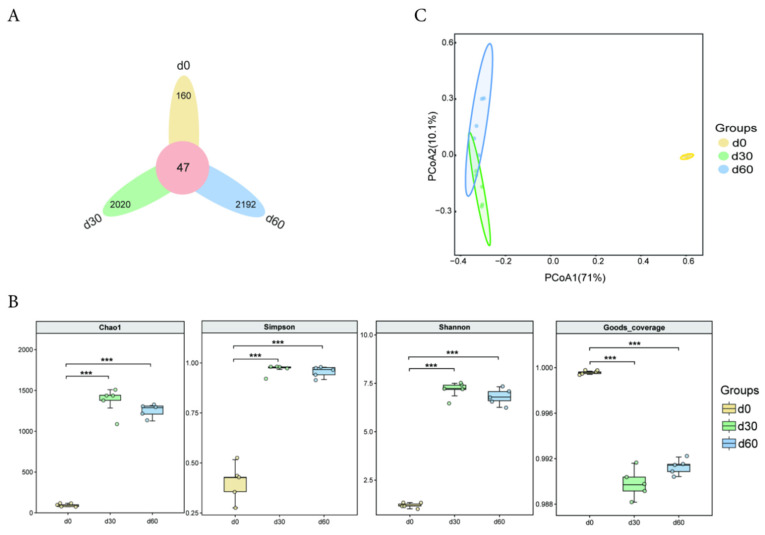
Diversity analysis of microbial communities in Jinmu grain grass silage. (**A**)—Venn analysis (ASV/OTU); (**B**)—Group box plot of α diversity index; (**C**)—PCoA two-dimensional sorting diagram; *** *p* < 0.01 vs. d0.

There were a total of 47 OTUs in the bacterial community during the ensiling process of Jinmu grain grass. The specific OTUs of the bacterial community after 0, 30, and 60 days of ensiling were 160, 2020, and 2192, respectively (Figure 1A). The rarefaction curve of each group of silage samples tends to flatten, indicating that the sequencing quantity of the samples in this experiment is reasonable, and the obtained communities can reflect most actual species (Figure S1). The Chao1, Simpson, and Shannon indices after 30 and 60 days of ensiling were extremely significantly higher than those at 0 days (*p* < 0.01), while the Goods coverage index was extremely significantly lower than that at 0 days (*p* < 0.01) (Figure 1B). The PCoA analysis results of the bacterial community of Jinmu grain grass silage are shown in Figure 1C. The bacterial communities at 0, 30, and 60 days were significantly separated, indicating that different bacterial community structures were constructed at different storage times.

#### 3.3.2. Microbial Community Structure and Differences

**Figure 2:**
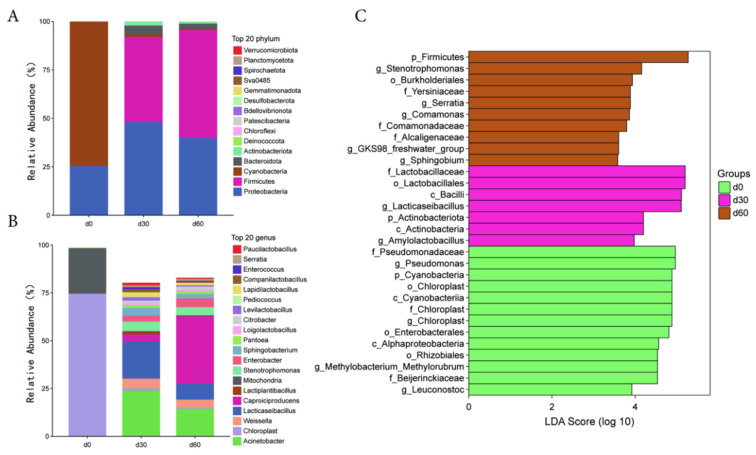
Analysis of microbial community structure and differences in Jinmu grain grass silage. (**A**)—bar chart of species composition at the phylum level; (**B**)—bar chart of species composition at the genus level; (**C**)—bar chart of LDA effect values of significant differences in microorganisms.

During the ensiling process, the main dominant bacteria phylum at 0 days are Cyanobacteria and Proteobacteria. The main dominant bacteria phylum at 30 and 60 days were Proteobacteria and Firmicutes (Figure 2A). The main dominant bacteria genus at 0 days were Chloroplast and Mitochondria. The main dominant bacterial genus at 30 days were *Acinetobacter*, *Lacticaseibacillus*, and *Weissella*. The main dominant bacterial genus at 60 days were *Caproiciproducens*, *Acinetobacter*, and *Lacticaseibacillus* (Figure 2B).

The LEfSe analysis of significantly different species in the microbial community of Jinmu grain grass silage is shown in Figure 2C. At the genus level, the relative abundance of *Pseudomonas*, *Chloroplast*, *Methylobacterium_Methylorubrum*, and *Leuconostoc* at 0 days were significantly higher than those of other storage times (*p* < 0.05); The relative abundance of *Lacticaseibacillus* and *Amylolactobacillus* after 30 days of ensiling were significantly higher than those of other storage times (*p* < 0.05); The relative abundance of *Stenotrophomonas*, *Serratia*, *Comamonas*, *GKS98_freshwater_group*, and *Sphingobium* after 60 days of ensiling were significantly higher than those of other storage times (*p* < 0.05).

### 3.4. The Influence of Different Storage Times on the Metabolomics of Jinmu Grain Grass

#### 3.4.1. Metabolomics Profile Analysis

**Figure 3:**
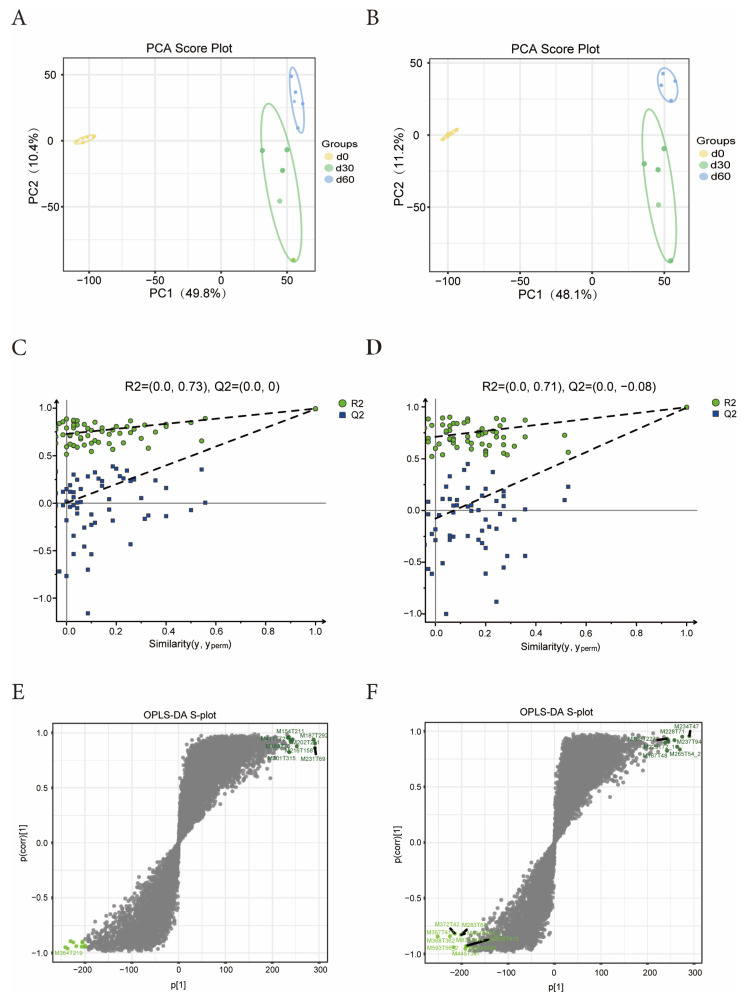
Effects of different storage times on microbial metabolism of Jinmu grain grass silage. (**A**)—positive mode PCA graph; (**B**)—negative mode PCA graph; (**C**)—positive displacement test plot; (**D**)—negative displacement test plot; (**E**)—positive S-plot; (**F**)—negative S-plot.

The PCA score graph analysis results show that under the positive and negative ion mode scanning, the samples within the same storage time can aggregate well, and the distance between each storage time is relatively long (Figure 3A,B), indicating that the metabolites of Jinmu grain grass have undergone specific changes with the extension of storage time. It can be seen from the PLS-DA displacement test plot that the R2 and Q2 points in the positive and negative ion modes are both lower than the original R2 and Q2 points in the upper right corner, indicating that the model has no overfitting and good predictive ability (Figure 3C,D). Under the OPLS-DA model between different storage times, R2Y was greater than 0.99 and Q2 was greater than 0.98, indicating that the OPLS-DA model had good stability and reliability. Most of the metabolite ions deviated from the origin, and the ions aggregated around the origin were in the minority (Figure 3E,F). It indicates that there are significant differences in the metabolites of Jinmu grain grass silage at different storage times.

#### 3.4.2. Analysis of Secondary Differential Metabolites After 0, 30, and 60 Days of Ensiling

**Figure 4:**
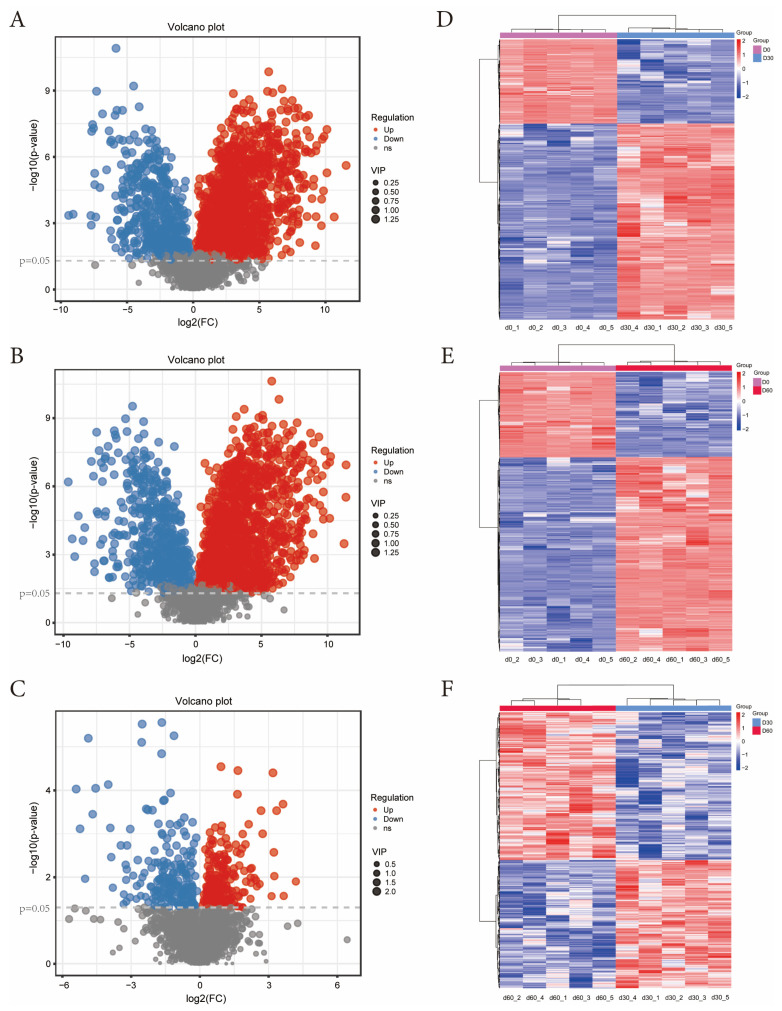
Volcano plot and clustering heat maps of secondary differential metabolites in each comparison group of Jinmu grain grass silage. (**A**)—Volcano plot of d30 vs. d0; (**B**)—Volcano plot of d60 vs. d0; (**C**)—Volcano plot of d60 vs. d30; (**D**)—Cluster heat map of d30 vs. d0; (**E**)—Cluster heat map of d60 vs. d0; (**F**)—Cluster heat map of d60 vs. d30.

Metabolites with *p* < 0.05, VIP > 1, and FC > 2 were selected, and a total of 2958 secondary identified metabolites were obtained. The volcano plot results of each comparison group of Jinmu grain grass silage show (Figure 4A–C) that compared with 0 days, 30 days of ensiling has 471 kinds of metabolites down-regulated and 1344 kinds up-regulated; Compared with 0 days, 60 days of ensiling has 522 kinds of metabolites down-regulated and 1344 kinds up-regulated; Compared with 30 days, 60 days of ensiling has 199 kinds of metabolites down-regulated and 219 kinds up-regulated; The clustering heat map results of each comparison group showed (Figure 4D–F) that the contents of differential metabolites after 30 and 60 days of ensiling changed significantly compared with 0 days (*p* < 0.05); Compared with 30 days, the content of differential metabolites after 60 days of ensiling also changed significantly (*p* < 0.05).

#### 3.4.3. Venn Analysis of the Secondary Differential Metabolites in Each Comparison Group

**Figure 5:**
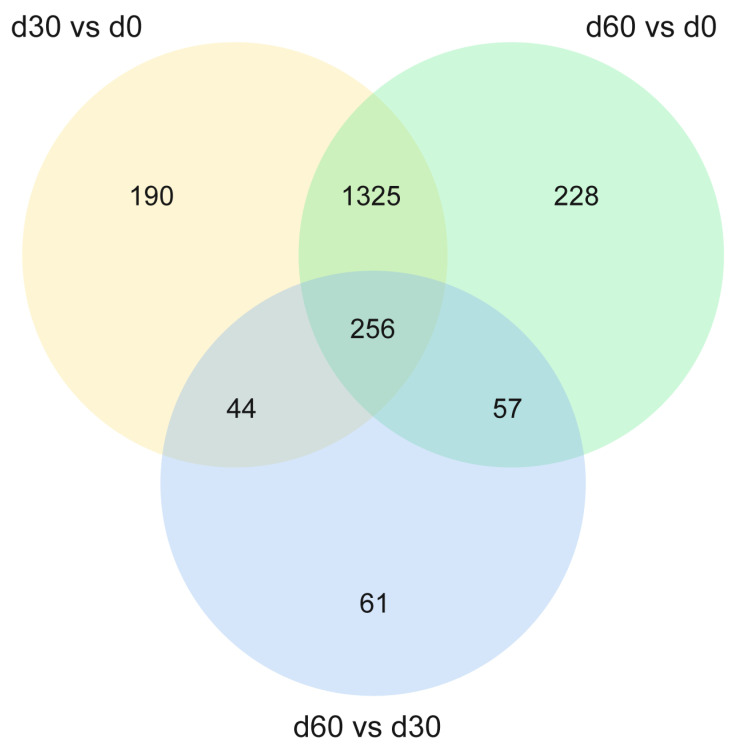
Venn analysis of differential metabolites among the comparison groups.

To further clarify the differential metabolites that play a key role in the ensiling process of Jinmu grain grass, Venn analysis was conducted on the secondary differential metabolites of each comparison group. The results showed (Figure 5) that there were 256 common differential metabolites (Table S1), mainly including amino acids, organic acids, fatty acids and their derivatives, which were significant changes in the three comparison groups (*p* < 0.05). Among them, compared with 0 days, both 30 and 60 days of ensiling have 78 kinds of metabolites down-regulated and 178 kinds up-regulated. Compared with 30 days, 60 days of ensiling has 98 kinds of metabolites down-regulated and 158 kinds up-regulated. It indicates that most metabolites gradually accumulate or consumed with the extension of storage time, and a few metabolites reached the highest content after 30 days of ensiling.

#### 3.4.4. Enrichment Analysis of KEGG Pathways for Differential Metabolites

**Figure 6:**
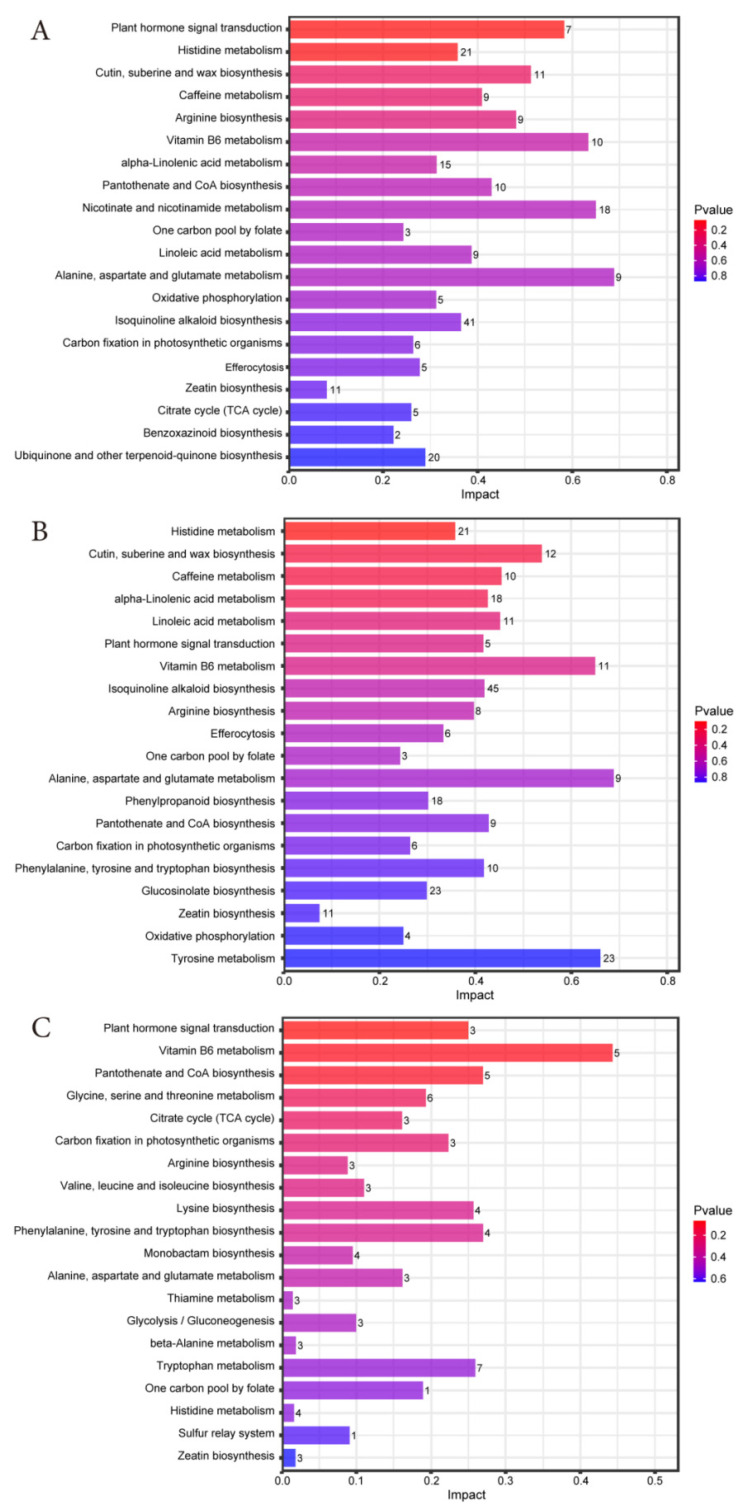
Bar chart of metabolic pathway influencing factors in each comparison group of Jinmu grain grass silage. (**A**)—d30 vs. d0; (**B**)—d60 vs. d0; (**C**)—d60 vs. d30.

Based on the criteria of impact > 0.1, and *p* < 0.05, we screened the top 20 metabolic pathways with significant effects in each comparison group between 0, 30, and 60 days (Figure 6A–C). The results showed that the common metabolic pathways of d30 vs. d0, d60 vs. d0 and d60 vs. d30 were plant hormone signal transduction, Histidine metabolism, arginine biosynthesis, Vitamin B6 metabolism, Pantothenate and CoA biosynthesis, one-carbon pool by folate, Alanine aspartate and glutamate metabolism, carbon fixation in photosynthetic organisms, and Zeatin biosynthesis. It indicates that the key differential metabolites of Jinmu grain grass silage are significantly enriched in these nine metabolic pathways. In addition, its metabolic pathways are mainly composed of amino acid metabolism, lipid metabolism, carbohydrate metabolism, and energy metabolism, involving phenylalanine tyrosine and tryptophan biosynthesis, alpha-linolenic acid metabolism, linoleic acid metabolism, glycolysis/gluconeogenesis, and citrate cycle (TCA cycle) and other metabolic pathways.

## 4. Discussion

### 4.1. The Influence of Different Storage Times on the Nutritional Composition and Fermentation Quality of Jinmu Grain Grass

It is generally believed that the nutritional composition of green forage is only about 10% lost when it is timely ensiled [16]. Through the analysis of experimental data, there was no significant difference in DM, CP, EE, NDF, and ADF after 0, 3, 7, 15, 30, and 60 days of ensiling (*p* > 0.05), while the RFV after 30 and 60 days of ensiling was significantly increased, indicating that the loss of nutrients during the ensiling process of Jinmu grain grass was relatively small.

During the ensiling process, a large amount of organic acids is produced, which rapidly lowers the pH value, inhibits the growth of harmful bacteria, and preserves the nutritional composition of the feed [17]. It is generally believed that the pH value of high-quality silage should be lower than 4.2. In this experiment, the pH value of Jinmu grain grass after 60 days of ensiling was 4.13, indicating that its ensiling effect was good. The degree of protein and amino acid decomposition in silage is usually reflected by the ratio of NH_3_-N to total nitrogen(TN). The larger the ratio, the more CP degradation occurs [18]. Under normal circumstances, if NH_3_-N for less than one-tenth of the TN, it indicates that the ensiling process is good. In this experiment, although the NH_3_-N/TN increased with the extension of storage time, the level remained below 10% all the time, and the change was within an acceptable range. After 60 days of ensiling, it only reached 5.08%, indicating that the ensiling process was well stored, inhibited the activity of microorganisms, and reduced the degradation process of CP. However, the rate of increase in the ratio during the experiment continued to accelerate, which might affect the nutritional value and palatability of the feed, and also increase the burden on the animal’s digestive system. This still requires further experimental verification.

Good silage should contain a relatively high amount of LA, a small amount of AA, and no BA. AA is produced by the metabolism of acetic acid bacteria and the decomposition of LA. In the early stage of ensiling, it can inhibit the reproduction of harmful microorganisms, but excessive use will affect the palatability of the feed [19]. The production of different acids during ensiling indicates that multiple microorganisms are engaged in fermentation, which can significantly reduce the pH value of silage and prolong the time of its aerobic transformation. The results of the organic acid content in this experiment showed that the LA content of Jinmu grain grass silage was all higher than the AA content, and BA was not detected in any of them. This indicated that Jinmu grain grass silage was of the lactic acid fermentation type. Moreover, the large amount of LA produced not only effectively inhibited other microorganisms but also improved the palatability and extended the shelf life of Jinmu grain grass silage.

Furthermore, it can be seen from the results of this experiment that the content of nutritional composition and fermentation quality of Jinmu grain grass both showed a continuous increasing or decreasing trend with the extension of storage time, indicating that it did not reach a stable state after 60 days of ensiling. This suggests that the storage time of Jinmu grain grass needs to be extended. Perhaps traditional ensiling methods are not suitable for Jinmu grain grass, but this still requires further research.

### 4.2. The Influence of Different Storage Times on the Microbial Community of Jinmu Grain Grass

At present, 16S rDNA sequencing technology is used to study the microbial community composition of various forage silages in order to better understand their raw material characteristics [20]. The experimental results of this study showed that Cyanobacteria and Proteobacteria were the dominant bacteria phylum at 0 days of Jinmu grain grass silage. It has been reported that the majority of the dominant bacterial phylum involved in ensiling belong to Proteobacteria and Firmicutes [21], and this study has reached the same conclusion. Proteobacteria and Firmicutes were the dominant phylum after 30 and 60 days of ensiling. At 30 days, the relative abundance of Proteobacteria was higher than that of Firmicutes. However, with the increase of storage time, Firmicutes surpassed Proteobacteria in relative abundance after 60 days of ensiling and became the dominant phylum. This was consistent with the study of Zhao et al. (2021) on alfalfa silage, which found that Firmicutes replaced Proteobacteria as the dominant phylum after 60 days of ensiling [22]. The dominant bacterial genus at 0 days of Jinmu grain grass silage was *Chloroplast* and *Mitochondria*, indicating that the initial microbial population mainly came from the plant materials themselves and the residual DNA of organelles. This result is consistent with the characteristics that plant-related microorganisms are still dominant when fresh forage is not ensiled [23]. At 30 days, the dominant bacterial genera were *Acinetobacter*, *Lacticaseibacillus,* and *Weissella*. Among them, *Acinetobacter* is a harmful bacterium that competes for oxygen consumption, delays the fermentation process, degrades proteins, and poses a risk of spoilage. The biogenic amines it produces, when ingested by animals, can affect their health. A few strains can degrade cellulose [24], and Jinmu grain grass is rich in cellulose. Whether *Acinetobacter* is a strain that degrades cellulose still needs further identification. As homolactic acid bacteria, *Lacticaseibacillus* and *Weissella* can rapidly produce LA to reduce the pH value and inhibit the growth of putrefactive bacteria [25]. This may be the reason why there is no spoilage in Jinmu grain grass silage when harmful bacteria are dominant, and it is also a sign of successful ensiling. At 60 days, the dominant bacterial genera were *Caproiciproducens*, *Acinetobacter*, and *Lacticaseibacillus*. *Caproiciproducens* replacing *Acinetobacte* as the most dominant bacterial genus may indicate that silage has entered the secondary fermentation stage. It generates volatile fatty acids such as caproic acid through LA conversion [26], further stabilizing the quality of silage. The continuous presence and relatively high abundance of *Acinetobacter* also indicate this point. Finally, *Lacticaseibacillus* is still exerting homologous fermentation and rapidly producing LA [25]. However, its relative abundance is lower than that of other harmful bacteria, indicating that Jinmu grain grass has entered the stable period after ensiling.

Through LEfSe analysis, differentially expressed species at the genus level with LDA_Score greater than 3.5 were selected and analyzed in combination with their relative abundance. The experimental results of this study showed that the relative abundance of *Pseudomonas*, *Chloroplast*, and *Leuconostoc* at 0 days of Jinmu grain grass silage was significantly higher than that at other times. Among them, *Pseudomonas*, as an aerobic putrefying bacteria, would decompose proteins and sugars under sufficient oxygen environment, produce ammonia gas and odor, increase pH value and harm early ensiling [27]. However, *Leuconostoc* produces LA, AA and carbon dioxide in the early stage of ensiling, which helps to establish an anaerobic environment and initial acidification, while inhibiting putrefactive bacteria and laying the foundation for the dominance of homolactic acid bacteria in subsequent fermentation. However, its characteristics as a typical heterolactic acid bacteria may delay the decline of pH value and lead to higher loss of DM [28]. With the extension of storage time, the abundance of some harmful bacteria in the early stage of ensiling began to decrease gradually, while the relevant bacterial genus promoting ensiling began to be significantly enriched [29]. At 30 days, Jinmu grain grass enters the middle stage of ensiling. The relative abundance of *Lacticaseibacillus* and *Amylolactobacillus* was significantly higher than at other times. During this fermentation stage, *Lacticaseibacillus* leads the isomorphic fermentation of LA, rapidly accumulating LA, reducing the pH value, inhibiting harmful spoilage microorganisms, and enhancing the stability of silage [25]. *Amyolactobacillus*, as a homolactic acid-producing lactic acid bacteria, can rapidly and efficiently utilize starch and soluble sugars to produce acid, significantly increasing LA yield and optimizing fermentation quality [30]. Although Jinmu grain grass has low starch content, the key role of *Amyolactobacillus* in accelerating acidification provides a stable foundation for the subsequent microbial community succession. At 60 days, silage enters the stable period. The relative abundance of microorganisms such as *Stenotrophomonas*, *Serratia*, and *Comamonas* was significantly higher than at other times. The enrichment of these pathogenic bacteria may be due to residual oxygen or pH value fluctuation in the late stage of ensiling. However, Firmicutes, which are significantly enriched at the phylum level at 60 days, can secrete a variety of enzymes, including cellulase and protease, as an important acid hydrolytic microorganism in an anaerobic environment [31], which can continuously ensure the stability of Jinmu grain grass silage. During the ensiling process, the dominant microbial community changes from Gram-negative bacteria to Gram-positive bacteria, and from pathogenic bacteria to lactic acid bacteria beneficial to ensiling. This might be due to the acidic environment in the later stage of ensiling being conducive to the growth of Firmicutes [32]. These differential microorganisms may all affect the nutritional value and fermentation quality of Jinmu grain grass silage. Therefore, it is necessary to conduct in-depth research on them.

### 4.3. The Influence of Different Storage Times on the Metabolomics of Jinmu Grain Grass

Through metabolomics analysis of 0, 30, and 60 days during the ensiling process of Jinmu grain grass, a total of 2958 secondary differential metabolites were detected in this study. It was found that the dynamic changes of metabolites reflected the specificity of the fermentation stage, indicating that the relative abundance of metabolites was significantly correlated with the silage time [33]. The volcano plots and clustering heat maps of each comparison group showed exactly the same trend with the change of storage time: The volcano plots showed that compared with 0 days, there are 471 kinds of metabolites down-regulated at 30 days, which is significantly lower than the 1344 kinds up-regulated. Meanwhile, the down-regulated metabolites after 60 days of ensiling slightly increased to 522, while the up-regulated metabolites were 1344, the same as those at 30 days. This result indicated that the metabolic activity at 30 days was more active, which may be related to the rapid proliferation of microorganisms and the decomposition of substrates. As the storage time was extended to 60 days, some metabolites showed a trend of continuous accumulation or consumption. In addition, compared with 30 days, the decrease and increase of metabolite quantity were similar at 60 days, indicating that the metabolism tended to balance in the middle and late stage of ensiling [34]. The clustering heat map showed that the content of differential metabolites changed significantly in all stages of ensiling, especially compared with 0 days. The overall difference of metabolites was more prominent after 30 and 60 days of ensiling. This indicates that the middle stage of ensiling is a crucial period for the accumulation or consumption of metabolites, which may be closely related to the fermentation activities of microorganisms such as lactic acid bacteria [35]. In addition, the changes at 60 and 30 days differential metabolites suggested that the late stage of ensiling may involve the formation or stabilization process of secondary metabolites, which was also consistent with the results of 16S rDNA sequencing analysis.

Through Venn analysis, a total of 256 common differential metabolites were identified that showed significant changes in all three comparison groups. These metabolites may be the key substances regulating the ensiling process. The results showed that compared with 0 days, both 30 and 60 days of ensiling had 78 kinds of metabolites down-regulated and 178 kinds up-regulated. Compared with 30 days, 60 days of ensiling has 98 kinds of metabolites down-regulated and 158 kinds up-regulated. It showed that most metabolites accumulated or consumed gradually with the extension of storage time, and a few metabolites reached the highest content at 30 days. These common differential metabolites mainly included amino acids, organic acids, fatty acids, and their derivatives. Among them, the metabolites such as 5-Aminolevulinic acid, aspartate semialdehyde, and pantothenic acid increased continuously at three times, indicating that protein degradation and energy metabolism were active during the ensiling process. The up-regulated differential metabolites may be involved in the core metabolic pathways of ensiling, such as glycolysis and amino acid metabolism. 5-Aminolevulinic acid, as a precursor for heme synthesis, its up-regulation may reflect the active regulation of nitrogen metabolism by microorganisms in the later stage of ensiling, promoting the transformation of chlorophyll degradation products, which is consistent with the phenomenon that the color of silage changes from green to yellow [36]. The accumulation of amino acid metabolites such as aspartate semialdehyde reflects the transformation and utilization of plant protein by microorganisms. Amino acids are the main metabolites produced by microorganisms through metabolic activities and are essential substances required by plants [37]. The up-regulation of aromatic amino acids may be related to the activation of amino acid metabolic pathways of microorganisms such as lactic acid bacteria, which can provide more abundant nitrogen sources for silage and may also affect the formation of flavor substances [38]. Pantothenic acid is involved in the biosynthesis of pantothenic acid and CoA. Its continuous up-regulation indicates that microorganisms in coenzyme metabolism are active during the whole ensiling process, which provides support for the rapid proliferation of microorganisms and substrate decomposition in the initial stage of ensiling, thus promoting the accumulation of LA and the decrease of pH value [39]. In addition, the content of a few metabolites such as Glyceraldehyde 3-phosphate reached the highest level at 30 days. As a key intermediate product in the glycolysis process, its significant accumulation may be related to the acidification stage dominated by lactic acid bacteria in the middle of ensiling [40]. Metabolites such as Fumaric acid and Homo-L-arginine continued to decrease at three time, indicating that the down-regulated differential metabolites might be related to the weakened microbial activity or substrate consumption in the later stage of ensiling. Fumaric acid, as an intermediate and a basic nutrient in the TCA cycle, its continuous consumption may indicate that the energy metabolism pathway slows down in the later stage of ensiling, and the utilization of carbon sources by microorganisms preferentially shifts to generating organic acids to lower the pH value [41], which is consistent with the transformation of the silage system from aerobic respiration to anaerobic fermentation. The reduction of Homo-L-arginine, as a key amino acid in the urea cycle, may result from the redistribution of nitrogen sources by microorganisms, such as its extensive use in the synthesis of bacterial proteins or conversion into other nitrogen-containing compounds [42]. These common metabolites provide potential markers for revealing the key regulatory nodes of Jinmu grain grass silage.

In recent years, with the development of bioinformatics technology, regulating metabolic pathways through the mechanism of microbiome has become a hot research topic. By regulating the metabolic pathway of microorganisms, the content of fermentation products can be increased and the fermentation can be promoted [43]. Among them, amino acid metabolism, lipid metabolism, carbohydrate metabolism, and energy metabolism have a significant influence on the material transformation in the ensiling process. Amino acid metabolism is converted into carbon dioxide and water in the tricarboxylic acid cycle, releasing energy, and plays an important role in plant protein synthesis and primary metabolism [32]. Lipid metabolism regulates the stability, nutritional value, and antioxidant properties of silage by influencing fatty acid degradation, flavor substance production, and microbial energy supply [44]. Carbohydrate metabolism provides energy for the life activities of lactic acid bacteria during ensiling; Energy metabolism is the most basic characteristic of plant metabolism, accompanied by energy release, transfer, storage, and utilization during the metabolic process [45]. These metabolic pathways play different roles in promoting ensiling of feed. In this study, the metabolic pathways of metabolites of Jinmu grain grass silage were mainly amino acid metabolism, lipid metabolism, carbohydrate metabolism, and energy metabolism. This result reflects that these metabolic processes, on the one hand, ensure the growth of microorganisms, and on the other hand, decompose the proteins, amino acids, lipids, and carbohydrates in silage, ensuring the improvement of fermentation quality while also resulting in a certain loss of nutritional composition.

The analysis of metabolic pathways showed that in the comparison between d30 vs. d0, d60 vs. d0, and d60 vs. d30, nine metabolic pathways such as plant hormone signal transduction, Histidine metabolism, and arginine biosynthesis, were enriched, indicating that these pathways played a core role in the ensiling process of Jinmu grain grass. The significant enrichment of amino acid metabolic pathways reflects the degradation of proteins and the utilization of amino acids by microorganisms during ensiling. Among them, Histidine metabolism and arginine biosynthesis are the core pathways of nitrogen metabolism in microorganisms, which affect the fermentation quality by regulating the amino acid balance. The metabolic products of Histidine, such as histamine, are involved in microbial stress responses and may be related to the acid tolerance mechanism of dominant bacterial communities such as lactic acid bacteria in the later stage of ensiling [46]. Arginine participates in nitrogen storage and release through the urea cycle, which affects the content of NH_3_-N/TN in silage [42]. In alanine, aspartate, and glutamate metabolism, glutamate is also the main entry point for microbial nitrogen assimilation. The α -ketoglutaric acid generated by its deamination will enter the TCA cycle for energy supply. The accumulation of alanine and aspartate may help microorganisms adapt to the low pH value environment of ensiling [47]. The activity of this pathway is consistent with the need to transition energy metabolism from the maintenance phase to the later stage of ensiling. Similarly, Vitamin B6 metabolism and Pantothenate and CoA biosynthesis are closely related to the energy metabolism and coenzyme synthesis of microorganisms, affecting ensiling efficiency and product accumulation. Among them, Vitamin B6 is a key coenzyme for amino acid metabolism and fatty acid metabolism, which supports the rapid proliferation of microorganisms and can also reduce the damage of reactive oxygen to plant and microbial cells in silage [48]. CoA is an essential factor for glycolysis, the TCA cycle, and fatty acid metabolism, which directly affects the degradation efficiency of carbohydrates in silage. It also participates in pyruvate metabolism, promotes LA fermentation, reduces pH value, and inhibits putrefactive bacteria [39]. In addition, the enrichment of plant hormone signal transduction may be related to the stress response and cell wall degradation of plant tissues during ensiling. Hormone signals participate in regulating the process of plant cell apoptosis in the early stage of ensiling, and at the same time, indirectly regulate the activity of attached microorganisms [49]. And Zeatin biosynthesis, as a sub-pathway of this pathway, is also involved in the processes of cell division and delaying plant senescence, affecting the retention of nutrients in the early stage of ensiling. The one-carbon pool by folate is connected to the methionine cycle, which may reduce the degradation loss of protein in silage. One carbon unit is the raw material for the synthesis of glycine, serine, purine, etc., and supports the rapid proliferation of microorganisms. The decrease of metabolites related to enzymes in carbon fixation in photosynthetic organisms reflects the decline of residual activity of plant photosynthesis, and the sugars formed by fixed carbon provide degradation substrates for subsequent fermentation, which is consistent with the complete entry of the silage system into the heterotrophic metabolism stage [50].

In addition to the common pathway, the metabolic changes of Jinmu grain grass silage were significantly enriched in amino acid metabolism, lipid metabolism, carbohydrate metabolism, and energy metabolism. Phenylalanine, tyrosine, and tryptophan biosynthesis can produce volatile phenolic substances, which affect the odor and antioxidant properties of silage. These three amino acids are all essential components for protein synthesis. This pathway may be inhibited in the early stage of ensiling due to the dominance of plant protein degradation, but it may become active again in the later stage dominated by microorganisms [51]. The activity of lipid metabolism may reflect the oxidation of lipids by microorganisms during ensiling, which is related to the preservation quality and nutritional value of silage [42]. Alpha-linolenic acid, as the main component of plant membrane lipids, may be oxidized by microorganisms during the ensiling process, which may be accompanied by changes in cell membrane permeability and promote the release of cellular contents to provide a carbon source for microorganisms [52]. Linoleic acid, as an essential fatty acid for ruminants, and its derivatives such as conjugated linoleic acid (CLA), can inhibit the growth of putrefactive bacteria and improve the stability of silage. The interaction between alpha-linolenic acid metabolism and linoleic acid metabolism may affect the fatty acid composition and oxidative stability of silage [53]. Glycolysis is the core pathway of the catabolism of carbohydrates. In the early stage of ensiling, lactic acid bacteria rapidly degrade carbohydrates into pyruvate and rapidly generate ATP, which are then converted into organic acids such as LA and AA to promote the decrease of pH value and the fermentation process, forming the initial stage of energy metabolism [54]. In the late stage of ensiling, some microorganisms may use LA or amino acids to re-synthesize carbohydrates through Gluconeogenesis to maintain metabolic balance. The TCA cycle is the core pathway of microbial energy metabolism, which completely oxidizes the pyruvate produced by glycolysis to generate a large amount of ATP and provide energy for microbial growth. The activity of the TCA cycle in the late stage of ensiling may reflect the continuous energy metabolism. Meanwhile, the TCA cycle works in synergy with glycolysis to promote the fermentation of heterologous lactic acid bacteria [54]. In addition, the TCA cycle serves as a hub for amino acid metabolism, with its key intermediates being precursors for the synthesis of amino acids such as glutamic acid and aspartic acid, thereby connecting the nitrogen metabolism network.

All the changes in metabolic pathways collectively reflect that during the entire ensiling process of Jinmu grain grass, it may be by altering the microbial community structure attached to the forage surface, regulating metabolic pathways, further changing the differential expression of metabolites, promoting the degradation of plant tissues, microbial metabolism, energy and material transformation, thereby better preserving the nutritional composition and improving the fermentation quality of silage [55].

Based on the Venn analysis and KEGG pathway enrichment analysis results of the secondary differential metabolites in each comparison group, we further clarified the interaction relationship between the microorganisms attached to the surface of Jinmu grain grass and their metabolites. We found that although the pathways for metabolite enrichment among the comparison groups between 0, 30, and 60 days were different, more metabolites were enriched in the amino acid metabolic pathways. Among them, Histidine metabolism, arginine biosynthesis, and alanine, aspartate, and glutamate metabolism were the pathways with the most co-enrichment of metabolites in the common metabolic pathways of each comparison group. The key differential metabolites are consistent with the performance of metabolic pathways, indicating that they are closely related to the metabolism of silage microorganisms. Previous studies have found that amino acid metabolism, carbohydrate metabolism, energy, and auxiliary factor metabolism are the main metabolic pathways related to silage corn fermentation [56]. Another study has shown that due to protein hydrolysis and sugar degradation during the ensiling process, amino acid metabolism and carbohydrate metabolism are the main microbial metabolic pathways affecting the flavor and quality of woody feed silage [57]. Our results are generally consistent with the above results. Amino acid metabolism accounts for a large proportion of the metabolic pathway in this experiment, and it participates in the regulation of material metabolism and information transmission of Jinmu grain grass silage. Amino acids are important components of proteins and polypeptides [58], and the changes of related differential metabolites in the fermentation process regulate the functional microorganisms of Jinmu grain grass silage. The growth and reproduction of microorganisms in silage are prone to protein hydrolysis, resulting in increased amino acid metabolism, thus affecting the protein content. The metabolic pathways of these amino acids may reflect the metabolic dynamics of the dominant microbial communities in Jinmu grain grass silage. Lactic acid bacteria, as the most important microorganisms in silage, have a significant impact on their metabolism and environmental tolerance due to the presence of amino acids. This might be the reason why amino acid metabolism accounts for a large proportion of Jinmu grain grass silage.

The application of microbiomics and metabolomics has enriched our understanding of silage microorganisms and metabolites and identified a variety of potentially beneficial metabolites. Furthermore, this study elucidated how the microbial ecosystem evolves to enhance the nutritional value of silage for livestock. It also clarified the relationship between key metabolites and fermentative bacteria, providing a theoretical foundation for expanding forage resources.

## 5. Conclusions

With the extension of storage time, the pH value of Jinmu grain grass silage decreased, and the contents of NH_3_-N/TN, LA, AA, and PA increased, indicating that silage can enhance its nutritional value. This experiment, based on 16S rDNA sequencing and comprehensive targeted metabolomics, revealed the changes in microbial communities such as *Acinetobacter*, *Lacticaseibacillus*, and *Weissella* in Jinmu grain grass silage, as well as metabolic pathways such as plant hormone signal transduction, Histidine metabolism, and arginine biosynthesis. Additionally, 256 differential metabolites common to all comparison groups were identified, including amino acids, organic acids, fatty acids, and their derivatives. The changes in these microbial communities and metabolites have promoted the preservation of silage nutrients and the improvement of silage quality. Therefore, Jinmu grain grass silage stands as an excellent forage resource for livestock and poultry. This study hopes to provide an important theoretical basis and technical support for the research on the fermentation mechanism of Jinmu grain grass silage and for expanding forage resources.

## Figures and Tables

**Table 1:** Nutritional composition contents of Jinmu grain grass silage.

Items	0 d	3 d	7 d	15 d	30 d	60 d	SEM	*p*-Value
DM	18.47	18.64	19.50	19.35	17.82	17.07	1.351	0.244
EE	10.45	11.05	10.74	10.69	10.54	10.54	1.326	0.750
CP	11.62	10.32	10.11	7.93	9.49	9.31	0.954	0.679
NDF	75.04	75.17	75.06	74.64	74.04	73.55	5.761	0.297
ADF	42.78	42.70	42.36	41.88	41.01	40.77	1.791	0.294
RFV	68.89 ^b^	68.85 ^b^	69.28 ^b^	70.14 ^b^	71.56 ^a^	72.27 ^a^	2.170	0.048

Note: Different letters in the shoulder marks of peer data indicate significant differences (*p* < 0.05), while the same letters or no letters indicate insignificant differences (*p* > 0.05). The same as Table 2. DM, dry matter; EE, ether extract; CP, crude protein; NDF, neutral detergent fiber; ADF, acidic detergent fiber; RFV, relative feeding value; SEM, Standard Error of the Mean, and the same as Table 2.

**Table 2:** Fermentation quality of Jinmu grain grass silage.

Items	0 d	3 d	7 d	15 d	30 d	60 d	SEM	*p*-Value
pH	6.16 ^a^	5.47 ^b^	5.06 ^bc^	4.51 ^cd^	4.33 ^de^	4.01 ^e^	0.093	<0.001
NH_3_-N/TN (%)	0.93 ^e^	3.07 ^d^	3.42 ^c^	3.89 ^bc^	4.32 ^b^	5.08 ^a^	1.033	<0.001
LA (%DM)	0.54 ^d^	1.07 ^de^	1.77 ^d^	2.94 ^c^	5.37 ^b^	6.57 ^a^	0.915	<0.001
AA (%DM)	0.29 ^d^	0.84 ^cd^	1.07 ^c^	1.12 ^c^	2.08 ^b^	2.77 ^a^	0.487	0.017
PA (%DM)	ND	0.04 ^b^	0.05 ^b^	0.06 ^b^	0.07 ^ab^	0.08 ^a^	0.006	0.001
BA (%DM)	ND	ND	ND	ND	ND	ND	-	-

Note: LA, lactic acid; AA, acetic acid; PA, propionic acid; BA, Butyric acid. ND means undetected.

## Data Availability

The original contributions presented in this study are included in the article and Supplementary Materials. Further inquiries can be directed to the corresponding author.

## Supplementary Materials

The following supporting information can be downloaded at: https://www.mdpi.com/article/10.3390/microorganisms13091973/s1, Figure S1: Rarefaction Curve of α diversity index of microbial communities in Jinmu grain grass silage; Table S1: Secondary key differential metabolites of Jinmu grain grass after 0, 30, and 60 days of ensiling.
