# Supplementary material for: Effect of Storage Time on the Fermentation Quality, Bacterial Community Structure, and Metabolic Profiles of Jinmu Grain Grass Silage
Source: Microorganisms. 2025 Aug 23;13(9):1973. doi: 10.3390/microorganisms13091973 (PMC12471645; doi:10.3390/microorganisms13091973)
Supplement: Supplementary file 1 [file microorganisms-13-01973-s001.zip › microorganisms-3815217-supplementary.pdf]

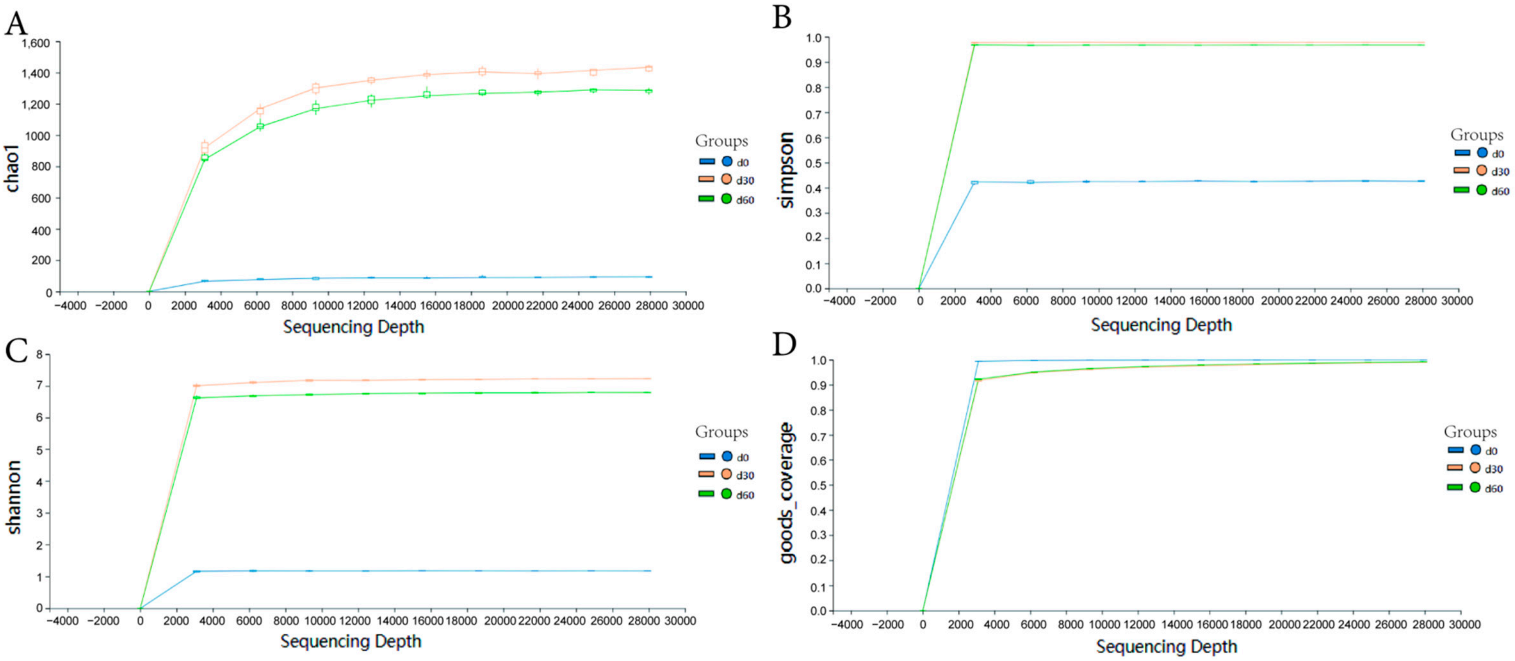

Figure S1. Rarefaction Curve of α diversity index of microbial communities in Jinmu grain grass silage.

(**A**)—Chao1 index; (**B**)—Simpson index; (**C**)—Shannon index; (**D**)—Goods coverage index

Table S1. Secondary key differential metabolites of Jinmu grain grass after 0, 30, and 60 days of ensiling

Compound	mz	rt	Relative abundance			Fold changes			Pos
			d60_Mean	d30_Mean	d0_Mean	d30 vs d0	d60 vs d0	d60 vs d30	neg
Glycyl-glycine	116.0347	52.5	50852228.56	13909739.7	8511506.23	1.63	5.97	3.66	pos
3-Methylbut-3-enoic acid	123.0408	42.4	578177580.3	320132921.9	174776462.8	1.83	3.31	1.81	pos
N-Acetylputrescine	131.1183	52.1	283568247.7	196526145.8	10531343.23	18.66	26.93	1.44	pos
N-Hydroxy-L-proline	132.1005	129.7	287658439.6	191737729.2	474518546.2	0.4	0.61	1.5	pos
4-Imidazolone-5-propionic acid	139.0508	68.9	1161558.7	3461801.65	173509.32	19.95	6.69	0.34	pos
Quinovose	147.1175	303	8084791.45	10436281.48	88428220.19	0.12	0.09	0.77	pos
Pyridoxamine	151.0872	68.5	2098653.45	453450.79	118648.73	3.82	17.69	4.63	pos
Styrene cis-glycol	156.1028	105.1	2551952.76	1174595.31	34700770.76	0.03	0.07	2.17	pos
4-Butyl-5-methylthiazole	156.139	281.3	63940586.41	24560859.32	12938645.39	1.9	4.94	2.6	pos
L-Threonine	158.1546	339	263174631.4	93459096.65	18671104.11	5.01	14.1	2.82	pos
2-Aminomuconic acid	158.0446	67.6	5708050.06	3238069.92	325074.17	9.96	17.56	1.76	pos
L-Kynurenine	165.1027	72.4	42515773.46	33971385.52	7703386.24	4.41	5.52	1.25	pos
2,3,4,5,6,7-Hexahydro-7-methylcyclopent[b]azepin-8(1H)-one	166.1234	139.9	11750351.12	9518451.2	4514367.78	2.11	2.6	1.23	pos
2,4-Undecadienal	167.1436	267	35871358.46	46167592.91	117876.8	391.66	304.31	0.78	pos
(+)-Iridodial	169.123	230	3905734.17	6733282.9	25470671.9	0.26	0.15	0.58	pos
8-Amino-7-oxononanoic acid	170.1185	239	52595342.19	46277552.55	17257863.54	2.68	3.05	1.14	pos
1-Hydroxy-2-naphthoic acid	171.044	42.5	49947529.95	13018820.57	5885844.94	2.21	8.49	3.84	pos
Campholenic alcohol	172.1341	251	234224473.6	179051941.5	17515044.19	10.22	13.37	1.31	pos
Chorismate	183.066	218.3	7641718.62	6250503.53	1363424.04	4.58	5.6	1.22	pos
1,6-didemethyltoxoflavin	183.066	259.5	15423504.65	19377860.91	3738178.25	5.18	4.13	0.8	pos
ACMC-20m7bf	187.1084	55.6	95393513.33	61379637.83	12704281.77	4.83	7.51	1.55	pos

Bufotenin	188.1116	55.9	3916431.33	2495212.24	1413837.74	1.76	2.77	1.57	pos
Homo-L-arginine	189.1283	248	85358486.64	59860819.92	147287872.7	0.41	0.58	1.43	pos
L-Formylkynurenine	193.0978	85	31597832.88	21946590.49	4754826.73	4.62	6.65	1.44	pos
2-Amino-4,6-dinitrotoluene	198.0535	85.5	1042726.27	3476682.81	14559268.2	0.24	0.07	0.3	pos
4-Chloro-7-nitrobenzofurazan	200.129	129.2	12475521.32	8246427.21	2075071.6	3.97	6.01	1.51	pos
Hexamethylene bisacetamide	201.1136	231.2	10453309.16	7712167.22	227633.91	33.88	45.92	1.36	pos
Dimethylguanidino valeric acid	202.1445	80.7	29180227.07	19677995.59	7764978.91	2.53	3.76	1.48	pos
2,4-diacetamido-2,4,6-trideoxy-alpha-D-man nopyranose	203.1398	93.7	14029634.89	141491135.2	1684007083	0.08	0.01	0.1	pos
Lysylthreonine	204.1352	43.5	297549.97	864327.62	33523960.68	0.03	0.01	0.34	pos
Heptyl butanoate	209.1542	220.3	3536301.88	4248893.81	2999018.73	1.42	1.18	0.83	pos
6-Benzylaminopurine	209.082	266.4	93920695.64	149589290.5	647884187.9	0.23	0.14	0.63	pos
Traumatic acid	211.1341	302.9	62746376.63	78385039.33	365046046.1	0.21	0.17	0.8	pos
Propyl 2,4-decadienoate	211.1702	361.8	18736333.62	12545050.48	2247299.96	5.58	8.34	1.49	pos
1,1-Diethoxy-2,6-nonadiene	213.0764	165.1	38763228.92	9553457.78	2628565.11	3.63	14.75	4.06	pos
Geranyl acetate	214.1812	253.1	11547151.65	30949296.25	1435076.28	21.57	8.05	0.37	pos
1-Isothiocyanato-8-(methylthio)octane	218.103	178.2	43667734.19	18358799.86	1491573.19	12.31	29.28	2.38	pos
N-Phenyl-2-naphthylamine	220.0998	258.2	50993670.04	26993253.54	5557870.32	4.86	9.18	1.89	pos
Pantothenic acid	220.1187	84.1	463543034	348572939.5	1975976.54	176.41	234.59	1.33	pos
Hydrocotarnine	222.0981	155.7	21231252.44	9975572.81	1062241.36	9.39	19.99	2.13	pos
10-Methyltridecanoic acid	229.1411	319.5	25938940.86	2544003	149094.31	17.06	173.98	10.2	pos
9,10-dihydrophenanthrene-9,10-diol	230.1872	70.3	3269162.39	10089037.84	434236291.7	0.02	0.01	0.32	pos
cis-N-methyl-alpha-berbine	233.0816	254.5	45454037.16	18024745.63	7527761.06	2.39	6.04	2.52	pos
Petasalbin	235.134	234.7	54801974.43	19807690.92	13527981.9	1.46	4.05	2.77	pos
Procaine	237.1246	84.3	76347629.22	36465558.09	22764008.51	1.6	3.35	2.09	pos
2-Carboxybenzalpyruvate	238.0721	224.3	21357646.15	33425449.54	143992057.4	0.23	0.15	0.64	pos

Chalciporone	244.1699	327.5	1979088.86	883869.53	66982292.41	0.01	0.03	2.24	pos
Methylgallic acid-O-sulphate	248.2463	423.3	8705936	13209976.73	20826033.43	0.63	0.42	0.66	pos
Caranine	255.124	271.2	15060131.54	12370580.79	1549791.75	7.98	9.72	1.22	pos
1-epi-Valienol 1-phosphate	257.1852	203.4	163655797.6	88895057.67	23559765.52	3.77	6.95	1.84	pos
(E)-10,11-Dihydro-alpha-atlantone	257.212	357.3	539324.33	2564790.87	197466.65	12.99	2.73	0.21	pos
4-Methoxybenzyl phenylacetate	257.1143	71	1053026.66	6023667.87	15742990.84	0.38	0.07	0.17	pos
Lysylleucine	260.1615	70.9	126624798.4	104112477.4	3385679.79	30.75	37.4	1.22	pos
L-beta-aspartyl-L-phenylalanine	263.1034	222.8	1254739.3	2005838.32	15761959.32	0.13	0.08	0.63	pos
benzyl-6-hydroxy-2-cyclohexene-on-oyl	264.1268	243.2	52967770.16	25669789.09	460086.86	55.79	115.13	2.06	pos
FA 4_2;O3	265.016	42.7	403763835.4	503955958.7	177543651.9	2.84	2.27	0.8	pos
Equilin	269.1398	237.7	13529915.68	23990666.48	5652455.6	4.24	2.39	0.56	pos
Cirsimaritin	271.0974	256.1	23888177.1	10977916.61	583621.02	18.81	40.93	2.18	pos
noroxomaritidine	272.1251	70.6	5909002.22	2750982.2	168107.57	16.36	35.15	2.15	pos
Tetramethylpyrazine	273.2074	271.5	52583116.57	26020616.32	1124098.32	23.15	46.78	2.02	pos
alpha-Terpinyl anthranilate	274.1768	74.5	59256478.42	44074303.03	737732.2	59.74	80.32	1.34	pos
Shogaol	277.182	368.7	3258957865	1868938164	79693921.21	23.45	40.89	1.74	pos
cis-Parinaric acid	277.1819	391.3	168738758.2	126464292	80465549.05	1.57	2.1	1.33	pos
4a-Carbinolamine tetrahydrobiopterin	278.1977	218.2	4357879.37	3399341.02	2117551.86	1.61	2.06	1.28	pos
Sphingosine	283.2832	343.3	13709385.47	8847781.69	21001.99	421.28	652.77	1.55	pos
Sphingosine(1+)	284.2673	423.4	406700971.1	539715055	361476665.2	1.49	1.13	0.75	pos
2'-Hydroxygenistein	287.0555	286.7	679849613.1	848891230.5	73720840.56	11.51	9.22	0.8	pos
Catechin	291.0874	227.4	1317494.85	836335.65	103369.28	8.09	12.75	1.58	pos
2,3-Dihydrothienamycin	292.1301	201.8	31514829.48	18070804.73	144022.91	125.47	218.82	1.74	pos
3,5-Dibromo-4-hydroxybenzoate	294.8616	221.3	934321.51	3121359.39	606117358.5	0.01	0	0.3	pos
Glutamylphenylalanine	295.1896	252.5	31696929.91	23317221.41	16334847.62	1.43	1.94	1.36	pos
Etamiphylline	297.2076	360.7	107183716.9	56288290.67	294998435.8	0.19	0.36	1.9	pos

SPB 18_2;O2	298.2747	331.5	5765509423	3117199160	1313174826	2.37	4.39	1.85	pos
p-Hydroxyphenethyl trans-ferulate	298.2467	408.2	464402648	278378695.1	59003205.41	4.72	7.87	1.67	pos
Vitamin A2 aldehyde	300.2038	153.7	11825880.23	9075070.6	584054.6	15.54	20.25	1.3	pos
16a-Hydroxyandrost-4-ene-3,17-dione	303.2175	280.4	72082528.01	43540794.31	10069459.55	4.32	7.16	1.66	pos
Isoformononetin	307.1657	117.4	17630633.15	5643182.3	53390667.79	0.11	0.33	3.12	pos
5-Deoxy-5-fluoroadenosine	308.1944	341.5	54355832.44	28883943.03	8472631.51	3.41	6.42	1.88	pos
4-Methylumbelliferone glucuronide	309.0971	234.7	20949958.99	31999099.72	5969114.42	5.36	3.51	0.65	pos
Picrocrocin	313.1632	224	40779868.24	27178970.78	205449995.2	0.13	0.2	1.5	pos
Herbacetin 4-methyl ether	317.0654	308.1	45892734.23	7193495.47	2825119.54	2.55	16.24	6.38	pos
Hydroxyprolyl-Tryptophan	318.1815	240.1	157876037.7	43338654.45	191634.48	226.15	823.84	3.64	pos
isopentenyl adenosine	318.1561	243.1	21760995.9	2290298.46	142766.98	16.04	152.42	9.5	pos
Dehydroferreirin	318.3011	313.1	22169730.5	29500029.87	328966639.1	0.09	0.07	0.75	pos
Promazine 5-sulfoxide	318.1662	71.8	51555706.22	64682056.22	780900329.1	0.08	0.07	0.8	pos
9alphaHydroxyandrosta-1,4-diene-3,17-dione	318.2027	71.8	96930892.55	24354254.19	3259407.25	7.47	29.74	3.98	pos
Leukotriene B4	319.2267	383.4	141321996.5	70852084.77	2221851.55	31.89	63.61	1.99	pos
Oripavine	320.1256	216.1	329984.05	1158219.6	41098987.34	0.03	0.01	0.28	pos
4,6-Docosanedione	321.3161	423.2	21963507.98	31188514.51	877681.09	35.54	25.02	0.7	pos
Corydalis L	324.1567	153.4	4480590.72	17067468.4	584406.56	29.2	7.67	0.26	pos
Salicin	325.2002	272.1	84705193.04	152118051	407012217.6	0.37	0.21	0.56	pos
6,8-Heneicosanedione	325.3102	416.9	15949298.53	36107614.54	887773.61	40.67	17.97	0.44	pos
4-Methylthiobutyl-desulfoglucosinolate	325.08	81	32085863.75	25965232.04	4448706.8	5.84	7.21	1.24	pos
Stanozolol	329.1687	297.2	6530658.18	3444569.11	1215269.97	2.83	5.37	1.9	pos
(-)-Bisdechlorogeodin	331.0815	304.4	1046729565	8615745687	85876318.22	100.33	12.19	0.12	pos
Gibberellin A34	332.1438	238.4	287483.35	995031.47	1694571.24	0.59	0.17	0.29	pos
gamma-Glutamyltryptophan	334.1409	215.6	268394.62	1358147.74	33461770.39	0.04	0.01	0.2	pos

7-Oxo-8,15-isopimaradien-18-oic acid	334.2954	300.9	6263647.15	10291703.11	138275189.1	0.07	0.05	0.61	pos
(ent-16betaOH)-16,17-Dihydroxy-9(11)-kauren-19-oic acid	335.1836	334.1	99117516.23	83854567.81	3761832.29	22.29	26.35	1.18	pos
MG(0_0_18_1(11Z)_0_0)	339.2902	434.1	19763379.17	7858339.96	46283057.77	0.17	0.43	2.51	pos
Pentamidine	341.1949	248.1	43992392.42	86568952.9	9746619.01	8.88	4.51	0.51	pos
10-Undecen-1-ol	341.2323	391.1	10180301.76	8325521.85	40831971.62	0.2	0.25	1.22	pos
8-HETE	343.2275	377.2	109967958.2	65412721.56	3460369.54	18.9	31.78	1.68	pos
2-Oxophytanate	344.3162	362.6	100271770.7	432719277.1	49729008.5	8.7	2.02	0.23	pos
6-Hydroxy-8-heneicosanone	344.2796	404.5	6920690	22876116.47	1263827.61	18.1	5.48	0.3	pos
3-Amino-3-deoxy-AMP	347.1266	248.3	34811316.89	23946900.11	5075375.77	4.72	6.86	1.45	pos
(2Z,6E)-3,7,11,15,19-Pentamethyl-2,6-eicosadien-1-ol	348.2748	304.6	15334211.41	58388273.45	366400009.6	0.16	0.04	0.26	pos
Gibberellin A53	349.1991	368.9	149858117	402488977.7	49483051.26	8.13	3.03	0.37	pos
Dhurrin	350.2028	314.9	97713253.7	47668658.8	5910840.16	8.06	16.53	2.05	pos
1-(1,2,3,4,5-Pentahydroxypent-1-yl)-1,2,3,4-tetrahydro-beta-carboline-3-carboxylate	350.2042	332.2	31682326.58	17770681.9	583307.35	30.47	54.31	1.78	pos
Deptropine	351.2435	211.3	27921350.57	39071528.44	163353413.1	0.24	0.17	0.71	pos
Isoandrocymbine	354.2171	251.7	3229809.69	5123450.1	1235140.27	4.15	2.61	0.63	pos
9S-HpETE	354.2633	386.9	4297201.33	1966282.72	7507419.15	0.26	0.57	2.19	pos
Lithocholic acid	359.2995	349.3	16918175.74	8140194.72	1314860.41	6.19	12.87	2.08	pos
10-Apo-beta-carotenal	360.2747	322.1	12126191.24	23313922.5	1796822.06	12.98	6.75	0.52	pos
Etoxazole	360.2519	363	7002206.01	3766611.18	13280676.89	0.28	0.53	1.86	pos
Margaroylglycine	366.3364	365.3	22536289.19	64085155.81	710666.24	90.18	31.71	0.35	pos
4,4'-Bis(2-hydroxyethylethylamino)-2,2'-dimethylazobenzene	368.2131	279.5	17474743.87	26025697.25	46497925.43	0.56	0.38	0.67	pos
Desmosterol	368.3159	428.3	51241851.13	14983297.97	3467434.16	4.32	14.78	3.42	pos

aklavinone	369.134	277.4	70749792.86	46285673.73	13467634.03	3.44	5.25	1.53	pos
SCHEMBL16431292	371.3168	393.9	213042408	132978471.4	1111422.32	119.65	191.68	1.6	pos
Prostaglandin F2b	372.3114	400.5	58420715.81	32504262.82	462874.82	70.22	126.21	1.8	pos
Junosidine	376.3079	326.6	27370423.76	60754432.03	111377655.3	0.55	0.25	0.45	pos
Ergosterol	380.3381	449.6	35959400.34	21081678.86	8087717.73	2.61	4.45	1.71	pos
Pantetheine 4'-phosphate	381.0805	52.1	109118845.2	62060179.06	724084155.8	0.09	0.15	1.76	pos
Colchicine	382.1717	72.1	55687007.68	27413598.96	2522561.79	10.87	22.08	2.03	pos
Mefruside	383.2169	410.3	2490898.4	4155411.09	39428187.84	0.11	0.06	0.6	pos
4'-Demethylepipodophyllotoxin	401.1236	301.7	33374926.68	16639974.23	69118615.5	0.24	0.48	2.01	pos
25-Hydroxycholesterol	403.1156	259.8	146797511.2	83801277.03	382895235.5	0.22	0.38	1.75	pos
Tomatidine	416.3526	328.9	5035261.58	753629.39	11129378.08	0.07	0.45	6.68	pos
Dihydrozeatin riboside monophosphate	417.1118	286	3675087.53	11271507.06	59301331.47	0.19	0.06	0.33	pos
(25R)-26-hydroxycholest-4-en-3-one	423.3232	400.6	348727.2	3173573.92	18643198	0.17	0.02	0.11	pos
Aloin	436.1687	271.6	4848879.98	7334627.57	73156346.22	0.1	0.07	0.66	pos
3betaH-glycyrrhetinate	453.3356	309	102192282.2	51179278.95	927014.89	55.21	110.24	2	pos
Ceanothenic acid	455.3515	394.2	56897328.57	25614547.53	5536195.97	4.63	10.28	2.22	pos
LysoPE(18_2(9Z,12Z)_0_0)	478.2899	382.5	298319186.4	64080261.17	33356048.48	1.92	8.94	4.66	pos
LysoPC(18_3(9Z,12Z,15Z)_0_0)	518.3304	362.8	98822.23	4171875.97	78757479.77	0.05	0	0.02	pos
3-hydroxyechinenone	550.4126	443.7	31777325.43	15745847.79	2239817.84	7.03	14.19	2.02	pos
Semi-beta-carotenone	551.2703	406	70706543.94	14728121.67	1650793.21	8.92	42.83	4.8	pos
2,11,20,29,37,38,39,40-Octazanonacyclo-tetracontane	553.4412	426.1	74977659.77	32758587.92	1381005.83	23.72	54.29	2.29	pos
PA(8_0_16_0)	559.4661	446.9	792312.9	1418942.59	7407671.62	0.19	0.11	0.56	pos
2-Octaprenyl-3-methyl-6-methoxy-1,4-benzoquinol	565.4898	403.2	378285207	115988883.9	20025003.91	5.79	18.89	3.26	pos
7,7',8,8'-Tetrahydrolycopene	579.5066	442.4	121955863.9	38462871.8	1846641703	0.02	0.07	3.17	pos

Dihydroisopentenyldehydrorhodopin	579.522	441.9	15022430.06	7515793.8	192271322.8	0.04	0.08	2	pos
Hydroxyspheroidenone	584.4564	384	2996663.3	835801.98	8812378.71	0.09	0.34	3.59	pos
Glacin A	597.5165	434.7	180369019.9	78792572.86	9404746.72	8.38	19.18	2.29	pos
Collettiside I	599.4009	222.8	7996368.44	28116174.42	53343073.47	0.53	0.15	0.28	pos
Momordicoside I	602.4227	444.7	110837605.3	51115416.76	7901265.44	6.47	14.03	2.17	pos
Diadinoxanthin	605.3999	223.7	4078353.03	2077688.52	708787.29	2.93	5.75	1.96	pos
11-alpha-O-beta-D-Glucopyranosyl-16alpha-O-methylneoquassin	607.3898	386.7	18954044.12	6637987.6	622830.23	10.66	30.43	2.86	pos
Caldariellaquinol	615.4736	443.7	15972114.11	26331420.23	6502512.15	4.05	2.46	0.61	pos
5-Amino-4-imidazole carboxylate	109.0044	448.2	2654260660	1469503852	47787794.64	30.75	55.54	1.81	neg
Hydroxymethylphosphonate	110.9855	45	289660.1	702333.04	19603141.59	0.04	0.01	0.41	neg
Fumaric acid	115.0038	45.6	5990560.38	7825536	425381849.1	0.02	0.01	0.77	neg
Aspartate semialdehyde	116.0356	48.1	72096500.92	45397119.61	2263661.17	20.05	31.85	1.59	neg
L-Aspartic acid	132.0304	45.9	868632424.9	641999860.7	182865539	3.51	4.75	1.35	neg
Adenine	134.0475	71.5	113716971.8	55560923.82	656538846.7	0.08	0.17	2.05	neg
Glyceraldehyde	135.03	46.8	490466717	601118670.3	185093847.9	3.25	2.65	0.82	neg
Urocanic acid	137.0358	54.9	4923217.64	6179323.44	1896892.04	3.26	2.6	0.8	neg
3-Nitrophenol	138.0198	49.2	78737033.92	98300983.31	34794496.06	2.83	2.26	0.8	neg
2-Deoxy-3-keto-scyllo-inosamine	142.0513	46.6	52467653.76	39699124.5	11350759.43	3.5	4.62	1.32	neg
2,4-Pentanedione	145.0509	50.7	26920680.06	18529171.53	77621695.16	0.24	0.35	1.45	neg
5-methylthiopentanaldoxime	146.0654	49.7	4019072.7	1313919.46	15685434.11	0.08	0.26	3.06	neg
(-)-trans-Carveol	151.0403	94.3	25371985.71	15957690.73	8276899.02	1.93	3.07	1.59	neg
1,2-Benzoquinone monoimine	152.0301	70	20234800.09	13809232.63	1726154.6	8	11.72	1.47	neg
2-n-Propyl-4-oxopentanoic acid	157.087	72.1	9793879.98	13531505.24	488396.62	27.71	20.05	0.72	neg
n-Butyl acetate	161.0822	53.1	71089643.68	122826163.9	853303.92	143.94	83.31	0.58	neg
3-Hydroxycinnamic acid	163.0406	71.6	266579048.7	196500918.3	13772479.56	14.27	19.36	1.36	neg

DL-Mannitol	164.0436	133.3	9633420.09	7440777.05	522584.85	14.24	18.43	1.29	neg
FAL 8_1	171.1025	237	66156506.27	43710402.07	27056.5	1615.52	2445.12	1.51	neg
meso-Diaminoheptanedioate	171.0777	51.5	785418.42	960785.62	5577580.12	0.17	0.14	0.82	neg
Glycylvaline	173.0935	52.3	13258596.1	33602260.99	7140454.52	4.71	1.86	0.39	neg
1,2,3-Propanetricarboxylic acid	175.0725	47.7	95038045.2	59563779.81	12585698.82	4.73	7.55	1.6	neg
3-Isopropylmalic acid	175.0611	76	11360288.67	29867808.02	166384.04	179.51	68.28	0.38	neg
5-Aminolevulinic acid	176.0566	47.6	46018352.17	34265493.9	3557749.69	9.63	12.93	1.34	neg
4-methoxycinnamic acid	177.0195	218.9	23333260.28	30794017.41	17260243.19	1.78	1.35	0.76	neg
3,5,6-Trichloro-2-pyridinol	177.9024	34.1	674754.7	559876.76	5008210.54	0.11	0.13	1.21	neg
(5S)-6-Hydroxy-5-isopropenyl-2-methylhexanoate	185.1184	227.9	3242446.03	5605812.46	70278750.14	0.08	0.05	0.58	neg
cis-1,2-Dihydro-3-ethylcatechol	185.0823	234.5	9167924.18	15167486.04	2454379.12	6.18	3.74	0.6	neg
(R)-Pelletierine	186.1133	95.2	482949093.7	636903630.4	53695578.11	11.86	8.99	0.76	neg
Ectoine	187.0728	49.5	36541699.56	28103276.76	4651945.06	6.04	7.86	1.3	neg
1-Isothiocyanato-6-(methylthio)hexane	188.0929	71.6	675606.01	2036599.07	106507715.3	0.02	0.01	0.33	neg
Permethric acid	188.9864	112.7	1586762.87	10653878.03	261264.97	40.78	6.07	0.15	neg
3-(3-hydroxybutanoyloxy)butanoic acid	189.0768	46.3	38489133.66	34215102.13	2603545.13	13.14	14.78	1.12	neg
Perseitol	193.0716	47.6	171405567.1	130175550.8	32624073.3	3.99	5.25	1.32	neg
p-Cymene	195.1027	79.7	2134841.79	3342569.66	8593313.57	0.39	0.25	0.64	neg
N-Acetylhistidine	196.073	50.2	1886329.79	17511509.95	51646.17	339.07	36.52	0.11	neg
1,3,4,6-Tetrachloro-1,4-cyclohexadiene	196.8855	41.5	6467513.03	4543221.01	582846.39	7.79	11.1	1.42	neg
Sebacic acid	201.1136	72.2	11506557.11	8272975.61	90099731.36	0.09	0.13	1.39	neg
Indolepyruvate	202.0515	223.3	7970765.92	3775094.55	45051.95	83.79	176.92	2.11	neg
6-Methoxymellein	207.0664	215.6	1403619.73	4519676.65	9415969.11	0.48	0.15	0.31	neg
L-2-Aminoethyl seryl phosphate	209.0306	45.6	2686350.7	3354331.38	8199115.03	0.41	0.33	0.8	neg
N-a-Acetyl-L-arginine	215.1151	52.3	442253.45	84764.95	6915871.41	0.01	0.06	5.22	neg

Glyceraldehyde 3-phosphate	214.9944	73.9	8575827.22	14710011.65	844844.68	17.41	10.15	0.58	neg
Ala-Glu-OH	217.0833	48.3	67953223.55	129165881.3	24415171.15	5.29	2.78	0.53	neg
(+)-Neomenthol	217.1447	86.6	11423614.74	3770275.91	187844.47	20.07	60.81	3.03	neg
7-methylthioheptanaldoxime	220.1016	142.7	58021377.19	38108940.11	6947424.3	5.49	8.35	1.52	neg
Naphthalene-1,2-diol	221.0455	123.1	9350145.29	4531611.63	848415.85	5.34	11.02	2.06	neg
Isofraxidin	221.082	325.1	1572715.81	986826.16	7799833.86	0.13	0.2	1.59	neg
DKDI	221.0124	47	32505394.46	116019898.2	15115120.11	7.68	2.15	0.28	neg
2-Hydroxy-4-hydroxymethylbenzalpyruvate	221.0455	49.1	6459227.6	3892520.31	67580.4	57.6	95.58	1.66	neg
Nicotine	223.0284	285.5	19581421.09	31545500.34	7115629.82	4.43	2.75	0.62	neg
5-Oxo-1,2-campholide	227.0925	71.5	38036670	15305746.32	126460621.5	0.12	0.3	2.49	neg
Pyridoxal 5'-phosphate	228.0093	120.3	11463616.42	6755920.03	1347705.33	5.01	8.51	1.7	neg
alpha-Ketoisovaleric acid	231.0872	241.4	1536396.18	935522.66	91426.32	10.23	16.8	1.64	neg
Pentalenate	231.1351	71.3	76551530.12	90316213.77	57179700.63	1.58	1.34	0.85	neg
Tabtoxinine-delta-lactam	233.0781	47.2	47678647.99	37734782.29	8257900.26	4.57	5.77	1.26	neg
Uridine	243.0622	69.8	1060578887	113138780.5	6444085.99	17.56	164.58	9.37	neg
Santonin	245.0486	161.9	62912938.16	49906899.69	266991.14	186.92	235.64	1.26	neg
Zederone	245.1396	97	661866879.5	1671847195	269277355.5	6.21	2.46	0.4	neg
Pentalenolactone F	259.0943	46.2	21310391.82	11713703.21	88215.61	132.78	241.57	1.82	neg
Amidinostreptamine	265.1201	74.3	12596798.91	11207408.33	3049438.29	3.68	4.13	1.12	neg
5-Hydroxybenzimidazole	267.09	235.6	2939867.2	2178101.52	198895.21	10.95	14.78	1.35	neg
Sotalol	271.061	247.2	4229857.31	333734.12	18901.15	17.66	223.79	12.67	neg
Trigonelline	273.088	132.7	469713.28	1161158.7	1711942.16	0.68	0.27	0.4	neg
FA 18_2;O	277.2177	440.3	653179905.1	1351316996	12317421359	0.11	0.05	0.48	neg
DMPP	299.0435	325.6	26134376.16	16756535.43	4082878.6	4.1	6.4	1.56	neg
Thiamine	310.114	52	2887042.27	7297389	631061.02	11.56	4.57	0.4	neg
methyl farnesoate	311.1852	295.3	806152082.4	538264020.6	4986323.95	107.95	161.67	1.5	neg

11a-Hydroxyprogesterone	312.1894	295.3	182335534.7	109020730	13378680.97	8.15	13.63	1.67	neg
gibberellin A20	314.2397	346.8	22009140.33	10989914.07	79857.11	137.62	275.61	2	neg
Tetrabenazine	316.1661	235.4	68230185.25	26326011.3	3439911.72	7.65	19.83	2.59	neg
Mercuron	319.9845	46.6	883852.58	1984150.83	20974207.54	0.09	0.04	0.45	neg
4[2-(5-Carboxy-2-hydroxy-3-methoxyphenyl)-2-oxoethylidene]-2-hydroxy-2-pentenedioate	333.0271	40.5	1784532.46	2705645.77	413178.67	6.55	4.32	0.66	neg
Diosbulbin B	343.1183	229	8588853.67	25752756.12	120913.69	212.98	71.03	0.33	neg
Pelanin	355.2491	298.6	14730786.52	11114351.89	831830.24	13.36	17.71	1.33	neg
12-Hydroxychelirubine	361.0689	212.7	1178911.23	752552.42	3729848.39	0.2	0.32	1.57	neg
3-O-Feruloylquinic acid	367.1065	77.7	27238062.57	19276337.74	5748665.53	3.35	4.74	1.41	neg
Tricetin Pentamethyl Ether	371.1691	183	32262231.47	17780687.81	185924.67	95.63	173.52	1.81	neg
6-Chloro-N-(1-methylethyl)-1,3,5-triazine-2,4-diamine	373.1164	214	27867295.42	14343779.82	2505779.66	5.72	11.12	1.94	neg
Dihydromacarpine	374.0989	209.2	599303.32	985288.12	2497469.08	0.39	0.24	0.61	neg
5-Deoxystrigol	375.1459	241.6	2475036.29	6576993.06	23131833.14	0.28	0.11	0.38	neg
4,7,13,16,21,24-Hexaoxa-1,10-diazabicyclo[8.8.8]hexacosane	375.191	245.1	19070267.69	11788756.11	3669923.36	3.21	5.2	1.62	neg
Glutathione episulfonium ion	379.0816	49.3	95623662.52	69696256.1	616614.92	113.03	155.08	1.37	neg
Asperlicin C	388.1115	272.5	6666132.46	4941712.95	450707.37	10.96	14.79	1.35	neg
7a-Hydroxy-cholestene-3-one	399.3314	334	36753.04	100818.9	1834029.87	0.05	0.02	0.36	neg
Harmolol	399.183	414.5	922411407.1	253025050.8	4866415.64	51.99	189.55	3.65	neg
LysoPA(16_0_0_0)	409.2351	349.6	6245173.14	9236150.84	90355979.94	0.1	0.07	0.68	neg
alpha-Tocotrienol	423.3262	430.2	166516949.2	110682828.2	2307928.76	47.96	72.15	1.5	neg
PHC 4-O-glucoside	431.0982	252.9	118744660.8	68508899.47	2789514.92	24.56	42.57	1.73	neg
Isohyodeoxycholic acid	437.1076	381.7	67910504.62	45784124.57	2791094.12	16.4	24.33	1.48	neg

5,10-Methylene-THF	439.131	221.8	2592276.52	862849.47	42387.51	20.36	61.16	3	neg
(3R)-3-hydroxy-16-methoxy-1,2-didehydro-2,3-dihydrotabersonine	443.133	306.9	21929721.42	47134044.36	8089185.22	5.83	2.71	0.47	neg
1,6-di-O-Galloylglucose	483.0684	264	1596028.98	4026395.42	48908052.11	0.08	0.03	0.4	neg
Pyridoxine 5'-phosphate	497.0794	353.3	69405485.55	56730875.76	15965414.39	3.55	4.35	1.22	neg
Ilexgenin A	501.3213	327.2	451251.95	2578883.66	89438.53	28.83	5.05	0.17	neg
Polyporusterone A	523.1944	274.5	360238.33	896927.48	3092373.39	0.29	0.12	0.4	neg
beta-D-Glcp-(1->4)-alpha-L-Rhap-(1->3)-D-Glcp	533.1796	315.1	177558296.9	45831733.18	4072367.62	11.25	43.6	3.87	neg
Spheroidene	567.4587	365.1	102340381.9	38788305.31	3189115.48	12.16	32.09	2.64	neg
L-Olivosyl-oleandolide	577.2803	319.7	12262593.15	28827400.34	50662768.54	0.57	0.24	0.43	neg
magnesium-protoporphyrin IX 13-monomethyl ester	579.1956	310.3	2804867.82	6525024.89	19740288.15	0.33	0.14	0.43	neg
all-trans-Retinoic acid	599.5238	414.4	283615488.2	396423332.9	84209156.76	4.71	3.37	0.72	neg
PA(16_0_16_0)	647.4741	418	3474850493	1766939439	181386160.4	9.74	19.16	1.97	neg
2-Octaprenyl-6-methoxy-1,4-benzoquinone	743.5288	415.5	82479231.21	32116027.39	4083145	7.87	20.2	2.57	neg
4-Hydroxy-2-methyl-3-oxo-4-farnesyl-3,4-dihydroquinoline-1-oxide	789.4731	433.2	221430.94	1323178.29	28557301.96	0.05	0.01	0.17	neg
2-methoxy-6-all trans-decaprenyl-2-methoxy-1,4-benzoquinol	801.6446	59.3	34151478.29	13968147.56	3569585.41	3.91	9.57	2.44	neg

Note: mz, mass-to-charge ratio; rt, retention time, in seconds; group_Mean, the average signal distribution of the metabolite across different groups; Fold changes(FC),the difference in the number of times metabolites varied between groups; posneg: the mode used to detect the substance, where pos denotes the positive ion mode and neg denotes the negative ion mode.
